# Adult stem cells at work: regenerating skeletal muscle

**DOI:** 10.1007/s00018-019-03093-6

**Published:** 2019-04-11

**Authors:** Manuel Schmidt, Svenja C. Schüler, Sören S. Hüttner, Björn von Eyss, Julia von Maltzahn

**Affiliations:** Leibniz Institute on Aging, Fritz Lipmann Institute, Beutenbergstrasse 11, 07745 Jena, Germany

**Keywords:** Satellite cell, Stem cell, Skeletal muscle, Regeneration, Myogenesis, Heterogeneity

## Abstract

Skeletal muscle regeneration is a finely tuned process involving the activation of various cellular and molecular processes. Satellite cells, the stem cells of skeletal muscle, are indispensable for skeletal muscle regeneration. Their functionality is critically modulated by intrinsic signaling pathways as well as by interactions with the stem cell niche. Here, we discuss the properties of satellite cells, including heterogeneity regarding gene expression and/or their phenotypic traits and the contribution of satellite cells to skeletal muscle regeneration. We also summarize the process of regeneration with a specific emphasis on signaling pathways, cytoskeletal rearrangements, the importance of miRNAs, and the contribution of non-satellite cells such as immune cells, fibro-adipogenic progenitor cells, and PW1-positive/Pax7-negative interstitial cells.

## Introduction

**Fig. 1:**
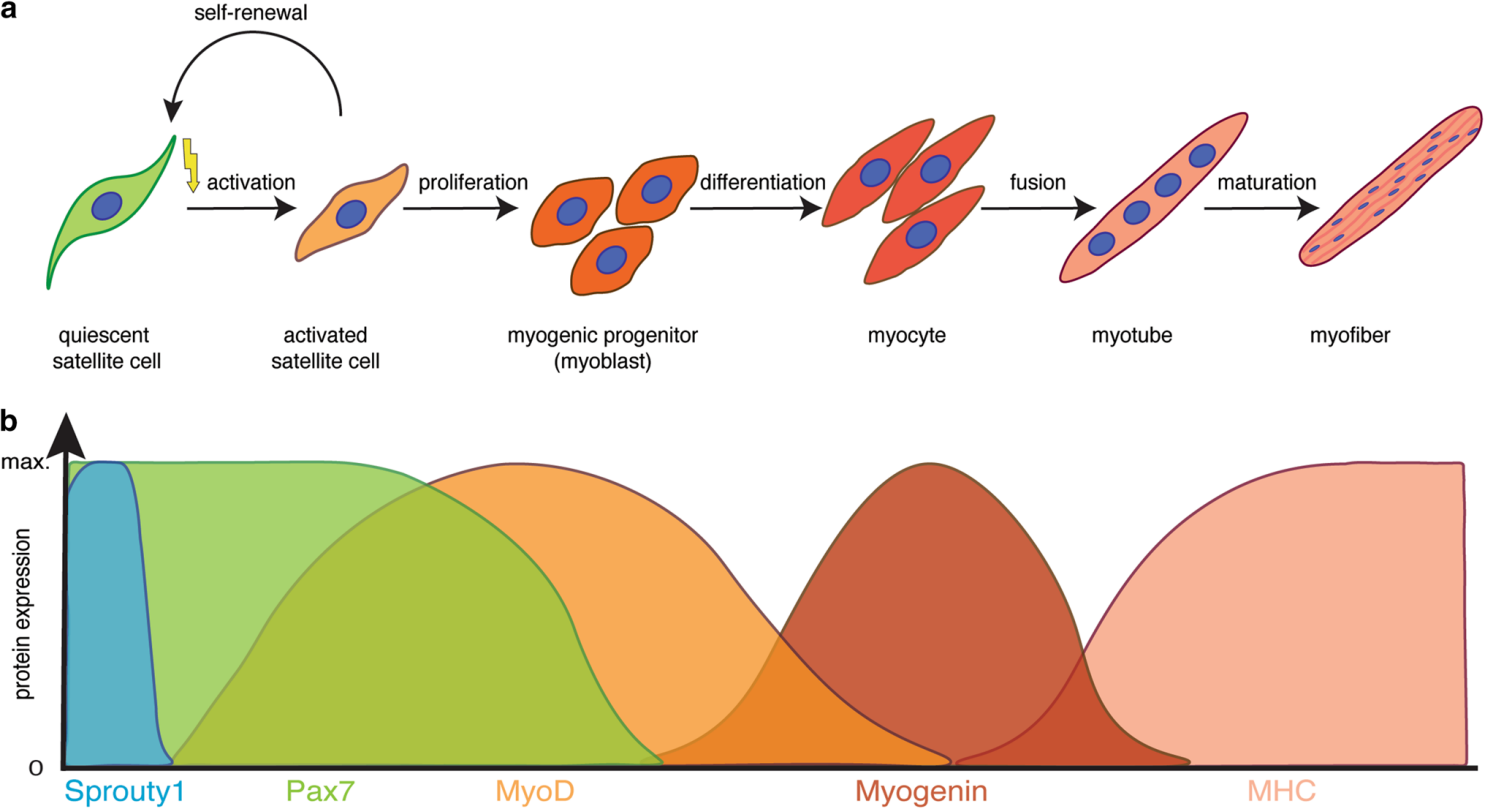
Myogenic lineage progression and expression profile of key myogenic regulators. **a** Schematic illustration of the myogenic lineage progression. Satellite cells are activated, e.g., due to injury, start to proliferate, thereby generating myogenic progenitor cells. Upon differentiation, myogenic progenitor cells differentiate into myocytes, which fuse to form myotubes and mature to become myofibers, the contractile unit of skeletal muscle. **b** Expression profile of key modulators of myogenic lineage progression

Skeletal muscle fulfils multiple functions in the body including voluntary locomotion, breathing, and postural behaviour. It possesses a remarkable ability to regenerate and to adapt to physiological demands such as growth or training [1]. Muscle stem cells—also termed satellite cells (SCs)—are a prerequisite for regeneration of skeletal muscle, as shown by previous studies using a diphtheria toxin (DTA)-based approach to deplete satellite cells [2–4]. Under resting conditions, satellite cells are quiescent and reside under the basal lamina of the myofiber [5]; this position, between the myofiber and the surrounding extracellular matrix (ECM), was responsible for their naming in 1961 by Alexander Mauro [6]. While quiescent under resting conditions, satellite cells become activated, expand and differentiate during skeletal muscle regeneration, a process controlled by sequential expression of transcription factors, resembling the differentiation program of embryonic myogenesis [1, 7] (Fig. 1). The paired box transcription factor Pax7 is expressed in all satellite cells, and is required for their postnatal maintenance and regeneration of skeletal muscle [8–10]. Upon activation, satellite cells co-express Pax7 and MyoD—an early marker for myogenic commitment— leave the quiescent state and further differentiate into myocytes, before maturing to myofibers. Notably, a subset of activated satellite cells downregulates MyoD and resists the differentiation process, thereby maintaining a mitotically inactive state similar to quiescence, a process depending on Sprouty1 [11–13]. Satellite cells have an enormous myogenic potential, which mostly depends on the expression of Pax genes and subsequent expression of myogenic regulatory factors (MRFs: MyoD, Myf5, Myogenin, and MRF4) [7]. Interestingly, ablation of satellite cells under homeostatic conditions in the adult does not seem to lead to muscle atrophy or myopathies without induced injury, suggesting that their main function in the adult is the regeneration of skeletal muscle [14, 15].

Although skeletal muscle can fully regenerate multiple times in healthy adult mice and men, the functionality of satellite cells declines in the course of various degenerative diseases or states such as aging resulting in impaired regeneration of skeletal muscle [16–21]. Such impaired functionality results from an altered extracellular matrix composition, misbalanced cell–cell interactions with residing cells of the skeletal muscle or changes of systemic factors such as loss of the anti-aging hormone Klotho [22–25]. It was recently demonstrated that soluble Klotho protein counteracts canonical Wnt3a signaling in satellite cells [22]. Importantly, Wnt3a signaling is upregulated in aged skeletal muscle, thereby impairing satellite cell function, resulting in disturbed regeneration [26]. The functional deficits of aged satellite cells can be partially overcome by systemic delivery of Klotho protein [27].

## Identification and characterization of satellite cells

Satellite cells were first characterized by their unique localization under the basal lamina of myofibers [6]. Recently, satellite cells have also been termed muscle stem cells (MuSCs), probably due to the fact that the term satellite cell also refers to specific glial cells in the brain. Satellite cells can self-renew, thereby maintaining the satellite cell pool, but also give rise to more differentiated myogenic progenitor cells, which then contribute to regeneration of myofibers after injury. These characteristics demonstrate that satellite cells are bonafide muscle stem cells. In adult skeletal muscle, all satellite cells express the paired box transcription factor Pax7, being essential for satellite cell function [8–10, 28], while subsets also express Pax3 [29] or myogenic regulatory factor 5 (Myf5) [30]. Other markers for satellite cells are located at the plasma membrane making them good candidates for satellite cell isolation via flow cytometry. Amongst others, these markers include α7-Integrin and β1-Integrin, M-Cadherin, Syndecan-4, Calcitonin-Receptor (CALCR), C-X-C Chemokine Receptor type-4 (CXCR4), Vascular Cell Adhesion Molecule 1 (VCAM1), and CD34 [31]. Of the genetic markers listed above, Pax7 is the canonical biomarker for satellite cells, since it is expressed in quiescent and activated satellite cells in most model species including mice, men, zebrafish, and chicken [32].

## Adult satellite cells: a heterogeneous cell population

The satellite cell population is heterogeneous. The individual subpopulations can be discriminated by different means, including gene expression or phenotypic traits such as division rate. Pulse-chase experiments using a Tg:Pax7-GFP mouse line demonstrate that satellite cells with a relatively high expression of Pax7 (Pax7^High^) are less primed for commitment and need a longer time to undergo the first mitosis compared to satellite cells with a relatively low expression of Pax7 (Pax7^Low^) [33]. Furthermore, those cells segregate their DNA asymmetrically, so that daughter cells receiving the template strand also maintain expression of stem cell markers [34, 35]. Another subset of satellite cells defined by phenotypic traits was identified and termed label-retaining cells (LRCs) using a TetO–H2B–GFP mouse line, in which administration of doxycycline marks rarely dividing or non-cycling cells by retention of the expression of the H2B–GFP reporter [36]. Further evidence for phenotypic heterogeneity within the satellite cell pool arises from a recent study by Tierney et al., who investigated the clonal dynamics of satellite cells following injury and found that, after multiple rounds of injury, the clonal complexity is reduced when using a multicolour lineage tracing approach [37].

Another approach for investigating satellite cell heterogeneity is based on genetic analysis using a Myf5-Cre reporter mouse line (R26R-YFP/Myf5-Cre). Here, approximately 10% of satellite cells have never expressed *myf5*, as demonstrated by the absence of the reporter YFP (yellow fluorescent protein) in Pax7-positive cells [30]. Interestingly, YFP^−^ satellite cells are able to engraft into the satellite cell niche after transplantation, while YFP^+^ satellite cells give rise to new myofibers and do not home to the satellite cell niche. However, upregulation of *myf5* and induction of the YFP reporter gene were demonstrated in YFP^−^ satellite daughter cells, further suggesting that Pax7^+^/YFP^−^ cells are a rare stem cell sub-population of satellite cells, while Pax7^+^/YFP^+^ cells are more committed progenitor cells [30]. Yet, following activation of satellite cells—for instance after injury—both populations of satellite cells (YFP^+^ and YFP^−^) are proliferating and undergo planar cell divisions, thereby generating two identical daughter cells (identical in terms of YFP expression). Multiple signaling pathways are driving the symmetric division of YFP^−^ cells, among them Wnt7a in concert with its receptor Fzd7 and the ECM molecule Fibronectin, as well as JAK/STAT signaling [17, 19, 38, 39].

## Asymmetric divisions of satellite cells

Asymmetric stem cell divisions are a prerequisite for proper stem cell renewal, concomitant with generation of progenitor cells. The first evidence for asymmetric satellite cell divisions emanated from a study by Shinin and colleagues. With the help of BrdU (bromodeoxyuridine) incorporation experiments, they demonstrated that the cell fate determinant Numb, a Notch signaling inhibitor, was segregated to the same daughter cell as the BrdU label during mitosis, suggesting a role for Numb in self-renewal [35]. Asymmetric satellite cell divisions are controlled by various signaling pathways, including Notch signaling [40]. Of interest is that Notch signaling seems to be responsible for asymmetric divisions of YFP^−^ satellite cells when using the R26R-YFP/Myf5-cre reporter mouse line, since the Notch effectors Notch3 and Delta1 are asymmetrically distributed in the daughter cells [30], the YFP^−^ satellite cells are expressing the Notch ligand Delta1, while the YFP^+^ satellite cells express the Notch receptor Notch3 [30]. The importance of Notch signaling for asymmetric satellite cell divisions is further supported by the fact that the Notch antagonist Numb is also asymmetrically distributed in different mouse models used to discriminate the different satellite cell subpopulations [34, 41, 42]. Besides asymmetric distribution of signaling molecules and receptors, DNA strands are also asymmetrically segregated during satellite cell division. The daughter cell retaining the template DNA strand shows more stemness characteristics, thereby attenuating the accumulation of replication errors in the parental DNA and the transmission to further daughter cells [33]. In addition, control of cell polarity plays an important role in the regulation of asymmetric satellite cell divisions. For instance, the Par complex, an evolutionary well-conserved complex located at the apical membrane, is a prerequisite for the asymmetric initiation of myogenic differentiation [43]. Activation of the Par complex leads to the selective activation of the p38α/β MAPK pathway, which, in turn, directly regulates MyoD transcription [44]. Dystrophin, a protein mostly known for its function in stabilization of the myofiber, is an essential cofactor for regulating cell polarity during asymmetric satellite cell division [45], suggesting that Duchenne muscular dystrophy is not only affecting the myofiber, but also the satellite cells. This is especially important, since skeletal muscle undergoes constant regeneration under dystrophic conditions, in both mice and humans.

## Regeneration of skeletal muscle

**Fig. 2:**
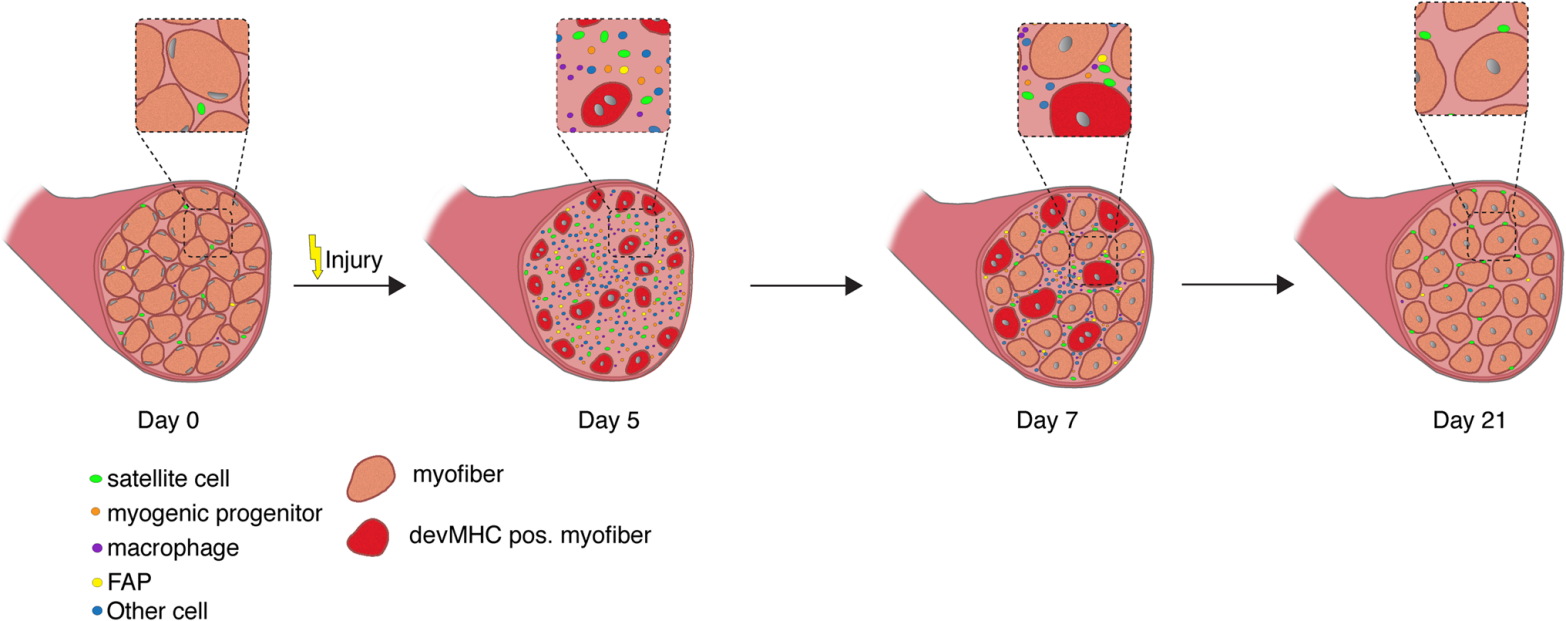
Regeneration of skeletal muscle. Time course of changes in cellular composition during skeletal muscle regeneration following cardiotoxin (CTX) injury. Satellite cells (in green) are quiescent in resting skeletal muscle. Five days after CTX injury, regenerating muscles are reduced to mostly mono-nuclear cells (satellite cells, immune cells, etc.), but are able to form new myotubes at day 7, which mature to multinucleated myofibers. Of note, the nuclei of intact myofibers are located at the periphery, while newly regenerating myofibers are characterized by centrally located myonuclei. During the maturation process, the myonuclei migrate to the periphery

The process of skeletal muscle regeneration can be divided into three phases: the inflammatory phase, the phase of satellite cell activation/differentiation phase, and the maturation phase, when remodeling of the newly formed myofibers occurs (Fig. 2).

The muscle degenerates after injury, starting with the necrosis of damaged myofibers. This is accompanied by an increased calcium influx and release of calcium from the sarcoplasmic reticulum of the damaged myofiber, leading to proteolysis and degeneration of the damaged tissue [32]. Inflammatory responses (phase 1 of regeneration) are triggered by necrosis of myofibers, including the recruitment of circulating leucocytes [32, 46]. The first inflammatory cells to be recruited to the damaged muscle are the neutrophils. Recruitment occurs within the first 6 h after muscle damage [47]. Subsequently, macrophages infiltrate the damaged muscle. The macrophage population consists of two distinct populations, the early macrophages infiltrating the muscle are the pro-inflammatory CD68^+^/CD163^−^ macrophages, followed by the anti-inflammatory CD68^−^/CD163^+^ macrophages [48–50]. The early infiltrating macrophages peak around 24 h after injury and are responsible for phagocytosis of damaged tissue parts and secrete pro-inflammatory cytokines such as TNFα and IL-1. The secondary wave of macrophages secretes anti-inflammatory cytokines such as IL-10 and is known to facilitate proliferation and differentiation of satellite cells [51–53]. Their greatest abundance can be observed 2–4 days after injury [25].

The second phase of regeneration of skeletal muscle is characterized by the activation and differentiation of satellite cells, a highly orchestrated process concomitant with morphological changes (Fig. 2). Quiescent under normal resting conditions, satellite cells are characterized by the expression of Pax7, but do not express MyoD [1, 54], although they may enter a G (alert) state priming them for rapid entry into the cell cycle, for instance in response to injury [55]. Upon injury, quiescent satellite cells enter the cell cycle and begin to express MyoD, migrate to the site of injury, and either fuse with damaged myofibers or become myogenic progenitor cells. Migration of satellite cells is controlled by signals from the myofibers, including signaling through Ephrin and Wnt7a [56, 57]. Myogenic progenitor cells are a highly proliferative transient amplifying cell population characterized by the expression of MyoD and Myf5, and are often referred to as myoblasts [5, 58]. The transcription factors Pax7 and Pax3 induce expression of genes responsible for promoting proliferation and commitment to the myogenic lineage and repress genes driving differentiation. The myogenic regulatory factors (MRFs) comprised of Myf5, MyoD, Myogenin, and MRF4 are downstream of Pax7 and Pax3, and promote myogenic differentiation [7, 59, 60]. Myogenin, a direct target of MyoD, initiates the terminal differentiation of myogenic progenitor cells, which is accompanied by downregulation of MyoD expression [1, 7, 60]. Morphologically, myogenic progenitor cells become elongated myocytes, which then fuse to form multinucleated myotubes. Newly formed myofibers are characterized by centrally located nuclei and the expression of devMHC (developmental myosin heavy chain), a myosin heavy chain which is, otherwise, only expressed during embryonic development [1, 32, 54]. This process is followed by maturation into myofibers (phase 3 of regeneration), which are the contractile units of skeletal muscle.

A highly orchestrated interplay between the stem cell niche and the satellite cell and other supporting cells is essential for proper regeneration of skeletal muscle. In conditions of perturbed homeostasis—such as aging—functional regeneration is hampered. Examples for changes in the satellite cell niche and changes in interactions with the satellite cell niche during aging include the loss of Fibronectin, altered β1-Integrin activity, or reduced levels of the anti-aging hormone Klotho, all of which result in impaired regeneration of skeletal muscle [22–24, 27]. A finely tuned balance between extrinsic cues and activation of intrinsic signaling pathways is required to accurately control satellite cell function. Multiple signaling pathways coordinate skeletal muscle regeneration. Below, a brief overview of how signaling pathways affect satellite cell function and regeneration of skeletal muscle is given.

## Wnt signaling during regeneration of skeletal muscle

**Fig. 3:**
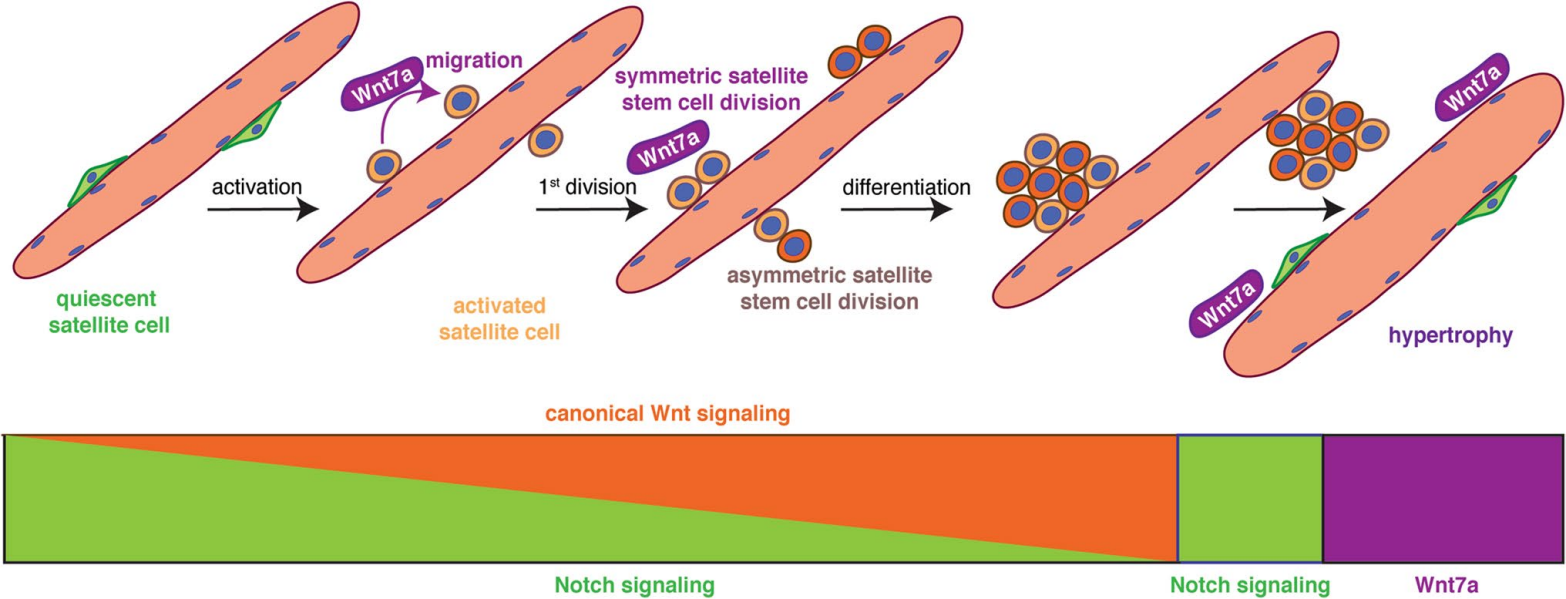
A switch from Notch to Wnt signaling is required for proper satellite cell differentiation. Satellite cells express high levels of Notch to retain them in a quiescent state, upon activation canonical Wnt signaling increases. The non-canonical Wnt7a drives the symmetric expansion of satellite stem cells and migration of satellite cells in general. Upon return to quiescence, satellite cells switch to Notch signaling, while Wnt7a drives the growth of already existing myotubes and myofibers, thereby inducing hypertrophy

Wnt signaling drives development of skeletal muscle and is one of the key signaling pathways involved in regeneration of skeletal muscle. [58]. Multiple Wnt ligands are expressed during regeneration of skeletal muscle in a temporally controlled manner. During the early phase of regeneration Wnt5a, Wnt5b, and Wnt7a are upregulated, while Wnt4 expression decreases. The later phases of regeneration are characterized by the expression of Wnt3a and Wnt7b [26, 61]. In adult skeletal muscle, canonical Wnt signaling—mainly through the ligand Wnt3a—drives differentiation of satellite cells, while non-canonical Wnt signaling through the ligand Wnt7a is responsible for promoting symmetric satellite cell divisions, migration of satellite cells, and growth of myofibers [26, 38, 39, 56, 62–65] (Fig. 3). In skeletal muscle, Wnt7a always signals through the Frizzled receptor Fzd7. Interestingly, several signaling pathways in satellite cells and myofibers are activated by the interaction of Wnt7a and Fzd7, namely the planar cell polarity pathway (PCP) and the AKT/mTOR pathway [58]. This makes Wnt7a a promising candidate for ameliorative treatment of muscle wasting diseases such as muscular dystrophy [66]. A recent publication describes the importance of R-spondin, a known modulator of canonical Wnt signaling for proper differentiation of myogenic progenitor cells during regeneration, further emphasizing the importance of proper Wnt signaling for regeneration of skeletal muscle [64, 67, 68]. The importance of balanced canonical Wnt signaling for regeneration of skeletal muscle is further highlighted by a recent study from Rudolf et al., who demonstrated that disruption or activation of β-catenin in adult satellite cells impairs the regeneration process [69].

## Notch signaling in satellite cells

A fine balance between signaling pathways and their precise activation is a prerequisite for effective regeneration of skeletal muscle—a good example is the temporal switch from Notch to canonical Wnt signaling required for proper differentiation (Fig. 3). Canonical Wnt signaling antagonizes the effects of Notch signaling, thereby allowing the progression of the myogenic commitment and differentiation [26]. The Notch receptor on the signal-receiving cell is expressed on the cell surface as a heterodimer, while the ligands are located on the opposing signal-sending cell. The Notch ligands are sequestered to the surface of the myofibers, thereby controlling satellite cell proliferation and differentiation [26, 40–42, 70]. The interplay between Notch and the transmembrane receptor Syndecan3 expressed in satellite cells controls the maintenance of the satellite cell pool and myofiber size after regeneration [71]. The importance of proper Notch signaling for satellite cell maintenance is further emphasized by two studies, demonstrating that expression of the downstream effector Recombination Signal Binding Protein for Immunoglobulin Kappa J Region (RBPJ) in satellite cells is a prerequisite for maintaining satellite cell quiescence [70, 72]. In mice lacking Notch1 and Notch2 in satellite cells, an inability to maintain quiescence can be observed, resulting in precocious entry into the cell cycle and in the end to a loss of satellite cells [73]. Cross talk of Notch signaling with the vascular niche is also important for regulating satellite cell quiescence—reminiscent of the role of cell adhesion molecules such as Cadherins, which control the transition from quiescence to activation through interaction with the myofiber niche [74, 75]. The importance of the interaction of Notch with the niche is further highlighted in a recent study by Baghdadi and colleagues who show that Notch-1/RBPJ controls expression of the extracellular matrix (ECM) molecule Collagen-V [76].

## Regulation of satellite cell function by miRNAs

miRNAs are controlling multiple signaling pathways in satellite cells. For instance, posttranscriptional regulation by miRNAs has been associated with maintenance of quiescence, activation, and differentiation of satellite cells [77, 78]. The biogenesis of miRNAs involves transcription by Polymerase II and several processing steps of the hairpin containing primary transcript that require the Ribonuclease III enzymes Drosha and Dicer to generate a double-stranded miRNA precursor. Loading of the miRNA precursor to Argonaute (Ago) protein family forms the RNA-induced silencing complex (RISC) that facilitates the final maturation towards a 22 nucleotide short single-stranded miRNA and recruitment to target messenger RNAs [79]. The binding of a miRNA to its target mRNA leads to inhibition of translation followed by mRNA de-capping, de-adenylation, and degradation [80]. The general importance of miRNAs in mouse development was demonstrated by a global Dicer deletion which results in embryonic lethality at embryonic day 7.5 (ED7.5) [81]. In the adult, genetic deletion of Dicer in Pax7-CreER mice revealed a critical role for miRNAs in regulating satellite cell quiescence. Dicer-deficient satellite cells leave quiescence and enter the cell cycle, but are also prone to cell death [82]. Although knowledge of miRNA function in satellite cells and their progeny remains limited, the functions of particular miRNAs have recently been unravelled.

About 351 miRNAs are differentially expressed when comparing quiescent and activated satellite cells, underscoring their importance for activation of satellite cells [82]. For instance, it has been shown that *myf5* mRNA is targeted by miR-31, which is highly expressed in quiescent satellite cells. In response to injury, miR-31 is quickly downregulated to allow a rapid translation of the Myf5 protein [83]. Furthermore, the cell cycle-promoting genes Dek, Ccnd2 (Cyclin D2), Cdc25a (Cell division cycle 25A), and Cdc25b1/2 (Cell division cycle 25B) are transcribed in quiescent satellite cells, but their translation is repressed by miR-489, miR-195, and miR-497 [82, 84]. Interestingly, transplantation of cultured satellite cells treated with miR-195/497 contributed more efficiently to regeneration of Dystrophin-deficient mice, suggestive of an increased ability to self-renew and to repopulate the stem cell compartment [84]. Surprisingly, only a few miRNAs have been identified, that are downregulated during differentiation, amongst them miR-125b targeting Insulin-like growth factor II (IGF-II) in myoblasts and miR-221 and miR-222 controlling cell cycle exit [85, 86]. Some microRNAs seem to be largely restricted to the skeletal muscle lineage and are, therefore, called myoMiRs. Of these, miR-1, miR-133 and miR-206 are muscle-specific miRNAs, which are strongly induced during differentiation. Their expression is directly regulated by the MRFs Myf5, MyoD, Myogenin, and Mef2, as well as Serum Response Factor (SRF) [87–90]. Expression of miR-1 is induced by MyoD and directly represses HDAC4 (Histon deacetylase 4), a negative regulator of skeletal muscle gene expression such as Mef2, thereby promoting myogenic differentiation [91]. Although miR-1 and miR-133 are transcribed as a bicistronic transcript, they exert different functions, e.g., miR-133 promotes proliferation of myoblasts by targeting SRF [91]. miRNAs control several signaling pathways important for satellite cell functionality. It has been demonstrated recently that components of the Sonic Hedgehog signaling pathway are controlled by miR-133, guiding the myogenic program during development [92]. Since the miR-206 has a very similar sequence to miR-1, it is assumed that they share the same targets. Consistently miR-206 also promotes differentiation in the myoblast cell line C2C12 cells by targeting HDAC4 [93]. The impact of miRNAs on cell fate decisions is further reflected by the function of miR-133 through the transcriptional regulator Prdm16 (PR domain containing 16), to regulate cell fate choices between the closely related muscle progenitors and brown adipocytes. Antagonism of miR-133 induces active brown adipocytes within regenerating skeletal muscle [94].

## Changes in the cytoskeleton of myogenic cells during regeneration

Cytoskeletal rearrangements are essential for proper regeneration of injured skeletal muscle. In resting skeletal muscle, quiescent SCs express high levels of α7- and β1-Integrin, which interact with the extracellular matrix (ECM) and regulate satellite cell fate [95, 96]. Furthermore, the large protein superfamily of Integrins is essential for migration, assembly of the ECM and is mostly associated with focal adhesion sites [97–99]. The importance of proper signaling via Integrins can also be appreciated by the fact that β1-Integrin (Itgb1) signaling is required for activation of satellite cells via the fibroblast growth factor 2 (FGF2) interaction in aged and dystrophic mice [24]. Vice versa, depletion of Itgb1 from satellite cells results in a phenotype resembling aging of satellite cells including deficits in self-renewal and functionality [20, 100, 101]. Integrin-mediated signaling is transmitted by focal adhesion kinases (FAK) at focal adhesion sites, whereas Integrin heterodimers themselves serve as an adaptor for connecting the ECM with the actin cytoskeleton [102]. Binding of FAK to Integrins leads to autophosphorylation of FAK at Tyr397, which, in turn, creates docking sites for other proteins such as Talin [103]. Besides Integrins, work on the Dystroglycan complex revealed important functions of Dystrophin and Dystroglycan in activated satellite cells and asymmetric satellite stem cell divisions in concert with Par1b [45]. In 2002, Cohn et al. demonstrated a functional role for Dystroglycan in satellite cell maintenance and self-renewal [104]. In addition, binding of members of the Dystrophin-associated Glycoprotein Complex (DGC) to Laminin induces the interaction of the small GTPase Rac1 with the DGC, particularly with Syntrophin [105, 106]. Rac activity is important for satellite cell migration by improving motility of satellite cells, thereby enhancing muscle strength after regeneration. Notably, Rac1 activity is also regulated by non-canonical Wnt7/Fzd7 signaling and induces the assembly of the mitotic spindle to drive asymmetric division by interaction with the Par complex [56, 107].

Several studies showed that remodeling of the actin cytoskeleton is pivotal for myoblast fusion and myotube formation, processes crucial for skeletal muscle regeneration [108–110]. FAK has an essential role in myoblast fusion and differentiation through interaction with β1-Integrin in Integrin clusters associated with Vinculin and the actin-binding protein Talin [111–113]. The adaptor protein Paxillin is subsequently recruited to focal adhesion sites [114–117], followed by recruitment of the actin-bundling protein α-Actinin, leading to an increased number of focal adhesion sites [118]. Similar to the actin cytoskeleton, several studies have revealed the importance of microtubule dynamics for the maintenance and formation of skeletal muscle also in human patients [119–121].

## Non-myogenic cells involved in regeneration of skeletal muscle

Although skeletal muscle regeneration is mainly driven by satellite cells and their ablation results in a failure to regenerate [2–4], several other cell types support the regeneration process. Those can be divided into two groups, those having myogenic potential such as myoendothelial cells, Pax7-negative Pw1+ interstitial cells (PICs) (PW1^+^/Pax7^−^ interstitial cells) and Twist2^+^ cells, contributing directly to the generation of new muscle fibers and those indirectly supporting regeneration. The latter group includes the immune cells and the Fibro-Adipogenic-Progenitors (FAPs).

As described above, immune cells are the first cells attracted to the site of injury in skeletal muscle. They are involved in preparation for regeneration by clearing the skeletal muscle environment from cell debris. To date, several types of immune cells have been shown to be important for proper regeneration of skeletal muscle, e.g., eosinophils, neutrophils, and M1 and M2 macrophages, with the eosinophils being the major source of IL-4 [32, 46]. Knockdown of IL-4 impairs regeneration of skeletal muscle by affecting the fate of FAPs, thus affecting regeneration indirectly [52]. Neutrophils are also recruited within the first hours after injury. Noteworthy, their depletion leads to severely impaired regeneration, since they are important for regulating macrophage function [46, 122–124], which are found in close proximity to blood vessels in resting muscle [125, 126]. Macrophages can be separated into two functional categories, M1 and M2 macrophages. The cytolytic activity of M1 macrophages is promoted by signals from the neutrophils [46, 122–124]. During disease, e.g., chronic infection, the switch from inflammatory M1 towards anti-inflammatory M2 macrophages is prolonged, contributing to impaired muscle regeneration [127]. Depletion of macrophages restrains clearance of necrotic tissue and hampers regeneration by impairing proliferation and differentiation of satellite cells [51, 128].

The other non-myogenic cells important for regeneration of skeletal muscle are the FAPs, which are located in the interstitium and can be identified by expression of PDGFRα (platelet-derived growth factor receptor-α). These are quiescent in healthy muscle, but proliferate upon injury. FAPs are a bipotent cell population, capable of differentiation into adipocytes and fibroblasts. Differentiation into adipocytes is controlled by different factors such as nitric oxide (NO) [129]. However, undifferentiated FAPs can have positive effects on activated myoblasts. In vitro and in vivo studies show that undifferentiated FAPs can induce differentiation of activated myoblasts by secreting molecules like IL-6, IGF-1, Wnt1, Wnt3a, and Wnt5a [130, 131]. In addition, FAPs also control satellite cell activation and proliferation in vitro in fiber culture assays [132]. The positive effect of undifferentiated FAPs is controlled by signals from intact muscle fibers under homeostatic conditions preventing differentiation of FAPs into adipocytes [131, 133]; while differentiation of FAPs into adipocytes is inhibited during regeneration by secretion of IL-4 by the eosinophils [52]. Besides differentiating into adipocytes, FAPs contribute to disturbed regeneration in disease or during aging by differentiating into fibroblasts, leading to increased fibrosis through secretion of collagen type I [131]. Interestingly, under disease or chronic injury conditions, more PDGFRα^+^ cells are present in skeletal muscle, with some of them already differentiated into myofibroblasts [134]. The increased number of myofibroblasts might be due to changes in the expression profile of macrophages. This is supported by the finding that macrophages express TGFβ (transforming growth factor beta), thereby inducing differentiation of FAPs into fibroblasts under disease conditions, while, in healthy muscle, apoptosis is initiated through expression of TNFα (tumor necrosis factor alpha) in macrophages [135, 136].

The group of non-satellite cells with myogenic potential includes myoendothelial cells, PICs and Twist2^+^ cells. PW1^+^/Pax7^−^ interstitial cells (PICs) were identified as a new population of cells having myogenic capacity [137]. In vitro studies showed that they start expressing Pax7/MyoD prior to formation of MHC-positive myotubes through fusion with other PICs or through fusion to satellite cell-derived myotubes [137, 138]. When transplanted into a regenerating muscle, PICs contribute to the formation of new muscle fibers at a level comparable to transplanted satellite cells [137, 139]. Not only do they contribute to muscle fiber formation during regeneration of skeletal muscle, but also secrete factors such as FGF-2 and IGF-1, known to promote satellite cell functionality [140]. Hence, PICs contribute to regeneration both directly and indirectly.

The Twist2^+^/Pax7^−^ cells are located in the interstitium of the skeletal muscle. In vitro analyses showed that Twist2^+^ cells lose Twist2 expression and start to express Pax7 when differentiation is induced. These cells are able to fuse with each other and with satellite cells in vitro. In vivo experiments further demonstrated that they contribute to regeneration of skeletal muscle by fusing to existing myofibers, as well as initiating myofiber formation. However, during embryogenesis, Twist2^+^ cells do not contribute to development of skeletal muscle [141].

## Concluding remarks and future perspectives

Satellite cells are the main drivers of skeletal muscle regeneration. The finely tuned balance between the states of quiescence, activation, and differentiation is a prerequisite for proper regeneration. Alongside cell intrinsic signaling, interactions with other cell types and the extracellular matrix play an important role in controlling these processes. In the future, the biggest challenge will be to gain a comprehensive understanding of how those processes interact and how they are altered in age and disease.
